# Functional benefit of joint surgery in patients with non-vascular Ehlers-Danlos syndrome: results of a retrospective study

**DOI:** 10.1186/s13023-024-03261-3

**Published:** 2024-09-23

**Authors:** Sharon Abihssira, Karelle Benistan, Geoffroy Nourissat

**Affiliations:** 1 Clinique Maussins-Nollet, COS Ramsay Santé, Paris, France; 2 AP-HP, Raymond Poincaré Hospital, Reference Center for Ehlers-Danlos Syndromes, Garches, France; 3 INSERM, UMR4179, University of Versailles-Saint-Quentin-en-Yvelines, Montigny le Bretonneux, France

**Keywords:** Ehlers-Danlos syndrome, Hypermobility, Shoulder, Wrist, Knee, Elbow, Ankle, Surgery, Joint laxity

## Abstract

**Background:**

Ehlers-Danlos syndrome (EDS) is a hereditary disease characterised by joint hypermobility, skin hyperextensibility and tissue fragility. Hypermobile EDS (hEDS is the more frequent subtype. Joint surgery may benefit certain patients after failure of medical treatments, but there is no consensus on the optimal surgical management of patients with hEDS. The aims of this retrospective study were to chart the surgical management of patients with hEDS, to determine the role of arthroscopy and to evaluate the functional results of joint surgery, including the reintervention rates.

**Results:**

A total of 69 patients with non-vascular EDS were evaluated (60 female; 87%). Mean (SD) age at first surgery was 25.6 ± 11.1 years. Among the 69 patients, first surgeries were carried out on the knee (*n* = 50; 39.4%), ankle (*n* = 28; 22.0%), shoulder (*n* = 22; 17.3%), wrist (*n* = 18; 14.2%) and elbow (*n* = 9; 7.1%). One-fifth of all first operations (20.8%) were carried out by arthroscopy, most often on the knee (36% of knee surgery cases). At the time of primary surgery, the surgeon was alerted to the diagnosis or suspicion of hEDS in only 33.9% of patients. The rate of reoperations (2 to ≥ 5) was 35.7% (10/28) for the ankle, 40.9% (9/22) for the shoulder, 44.4% (4/9) for the elbow, 50% (9/18) for the wrist and 60% (30/50) for the knee. Local or regional anaesthesia was badly tolerated or ineffective in 27.8%, 36.4% and 66.6% of operations on the wrist, shoulder and elbow, respectively. Overall, the majority of patients (> 70%) were satisfied or very satisfied with their surgery, particularly on the non-dominant side. The lowest satisfaction rate was for shoulder surgery on the dominant side (58.3% dissatisfied).

**Conclusions:**

Surgery for joint instability has a greater chance of success when it is carried out in patients with a known diagnosis of EDS before surgery. The majority of patients were satisfied with their surgery and, with the exception of the knee, there was a low rate of reoperations (≤ 50%). Arthroscopic procedures have an important role to play in these patients, particularly when surgery is performed on the knee.

## Background

Ehlers-Danlos syndrome (EDS) is a spectrum of heterogeneous genetic diseases caused by anomalies in the biosynthesis or structure of proteins integral to the extracellular matrix resulting in joint hypermobility, skin hyperextensibility and tissue fragility [1–3]. The disease has an estimated prevalence of 1/5000 live births and can affect males and females of all racial groups and ethnicities [3].

In 2017, the international classification of EDS described 13 different subtypes, of which 12 have a recognised associated genetic mutation [4]. A rare 14th subtype was described by Blackburn et al. in 2018 [5]. The majority of patients with EDS (80‒95%) are diagnosed with the hypermobile subtype (hEDS) [6], but this is the only subtype without an identified genetic cause [1].

EDS is characterised by musculoskeletal complications linked to joint instability and hypermobility. These include subluxations, dislocations, sprains, soft tissue lesions, and later complications such as tendonitis, tendon ruptures, muscle and ligament tears, muscle tension and spasms, osteoarthritis and chronic joint pain [7]. Patients with EDS are also more likely to suffer from cutaneous manifestations such as skin hyperextensibility and skin fragility [7]. In addition to joint hypermobility and skin problems, patients with EDS may experience gynaecological, ocular, oral, cardiovascular, gastrointestinal and neurological problems [7], fatigue and chronic pain [8, 9].

The diagnosis of EDS is based on clinical criteria [4] and is confirmed by molecular biology for all EDS subtypes except hEDS. The diagnosis of hEDS depends on the simultaneous presence of three criteria: (i) generalised joint hypermobility: a Beighton score ≥ 5 (adjusted according to age); (ii) the presence of systemic manifestations of a generalised connective tissue disorder and/or positive family history, with one or more first-degree relatives independently meeting the current diagnostic criteria for hEDS, and/or musculoskeletal complications; and (iii) the exclusion of other types of EDS or alternative diagnoses [4]. Hypermobile EDS appears to be autosomal dominant in transmission and predominantly affects females [10].

There is no cure for EDS and current approaches to treatment include physical therapy to improve joint stability, orthoses or compression garments, pain relief, appropriate treatments for other symptoms and psychological counselling [11]. Surgical treatment may be an option in specific patients after failure of medical treatments, particularly procedures for joint instability or nerve decompression. In 2020, Hoemere et al. reported the results of surgical procedures on the shoulder or knee in patients with hEDS/joint hypermobility syndrome (JHS) [12] and concluded that surgical management of shoulder or knee instability in these patients is challenging, probably due to the inferior biomechanical properties of the connective tissue. To date, there has been no consensus on the optimal surgical management of patients with EDS.

The aim of the current study was to chart the surgical management of patients with non-vascular EDS, to determine the place of arthroscopy and to evaluate the functional results of surgery, including the reintervention rate.

## Methods

### Study design and study population

This retrospective, descriptive, non-interventional study was carried out on a cohort of patients with a confirmed diagnosis of EDS and followed in the Reference Centre for Non-Vascular Ehlers-Danlos syndrome (NV-EDS) located in the Raymond Poincaré hospital, in Garches, France. The study was approved by the local Ethics Committee (CPP: n°2020-A0080831).

### Study population

The inclusion criteria were: patient ≥ 18 years of age, with a confirmed diagnosis of EDS (according to the 2017 classification of EDS) and having undergone surgery on at least one of the following joints: shoulder, elbow, wrist, knee or ankle. Patients were excluded if they were < 18-years of age, or if they had undergone surgery on the spine or hip as these joints were not evaluated in this study.

### Study aims

The main aim was to analyse the functional results of surgery on the shoulder, wrist, knee, elbow and/or ankle due to instability linked to EDS. The secondary aims were to determine: (i) the number of patients with a diagnosis of EDS before surgery; (ii) the number of patients undergoing reinterventions; (iii) the complications linked to anaesthesia and surgery; (iv) the relapse rates; and (v) the overall benefits of surgery.

The following criteria were analysed for each joint: (i) postoperative pain scored according to the visual analogue scale (VAS; score 0‒10, where 0 = no pain and 10 = worst pain imaginable); (ii) functional treatments received preoperatively (physical therapy, orthoses, compression garments); (iii) type of anaesthesia; (iv) peri- and postoperative complications; (v) reintervention rates.

The joints were assessed using joint-specific functional scores. The 11-item Quick-DASH score was used for the wrists and elbows, measured on a 5-point Likert scale where score = ([(sum of n responses)/n] -1)(25), with a higher score indicating greater disability; Subjective Shoulder Value (SSV) scored from 1‒100%, where 100% represents a normal shoulder; Lysholm-Tegner score (0‒100) for the knee, where 0 = worse disability and 100 = no disability; and European Foot and Ankle Society (EFAS) score for the ankle [13].

### Data collection

Demographic and clinical data were collected using a questionnaire compiled by a geneticist (KB) and an orthopaedic surgeon (GN). Each patient replied to the questions during a video consultation (SA).

### Statistical analysis

A descriptive analysis of the data collected in the questionnaires was performed. Quantitative data are expressed as the mean, standard deviation (SD) and median. Qualitative data are expressed as number and percentage. All calculations and analyses were carried out using SAS® software.

## Results

### Study population

**Fig. 1:**
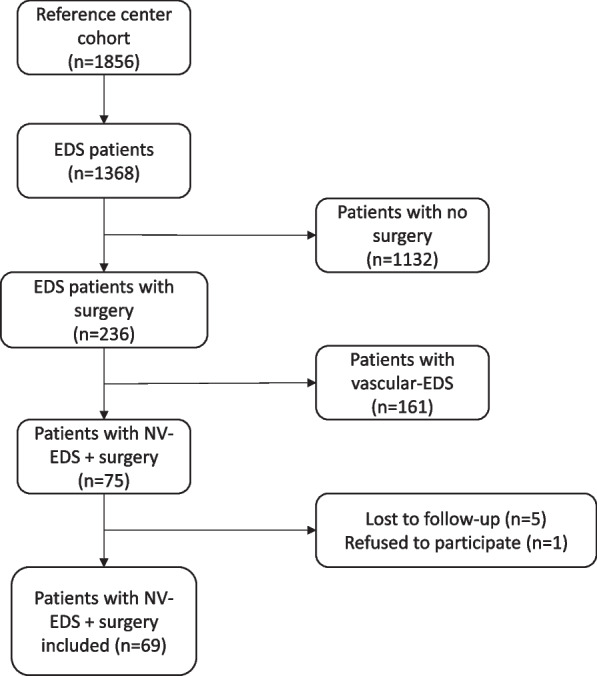
Flow chart for the study population according to 2017 EDS criteria

A total of 1856 patients with NV-EDS were followed in the reference centre and 75 of these were diagnosed with EDS according to 2017 criteria [4] and underwent surgery on one of the joints included in the study. Sixty-nine of these patients were included in the final analysis (60 female; 87%) (Fig. 1). Mean (± SD) age of the patients at inclusion was 40.7 ± 12.5 years. Mean time from primary surgery to inclusion was 200.1 ± 138.3 months. Among these patients, 55 had hypermobile EDS and 14 had others sub-types of non-vascular EDS.

The demographic characteristics of the study population are summarised in Table 1.

**Table 1:** Demographic characteristics of the study population (*n* = 69)

**Characteristic**	
Age (years)	40.7 ± 12.5
Age at primary surgery (years)	25.6 ± 11.1
Sex	
Female	60 (87%)
Male	9 (13%)
Dominant side	
Right	61 (88.4%)
Left	8 (11.6%)
BMI (kg/m^2^)	24.7 ± 6.1
Active smoker	16 (23.2%)
Employment	
Student	7 (10.1%)
Employed	27 (39.1%)
Disabled	17 (24.6%)
Sick leave	7 (10.1%)
Retired	5 (7.2%)
Job seeker	6 (8.7%)
Associated medical history^a^	
Ophthalmological and ENT	60 (87%)
Hepato-gastroenterological	57 (82.6%)
Genito-urinary	49 (71%)
Neurological	40 (70%)
Cardiovascular	31 (44.9)
Pulmonary	27 (39.1%)
Allergies	26 (37.7%)
Infectious diseases	25 (36.2%)
Endocrinological	21 (30.4%)
Renal	17 (24.6%)
Haematological	14 (20.3%)

Values shown are mean ± standard deviation or n (%)

*BMI* body mass index, *ENT* ear, nose and throat

^a^Only conditions with an incidence of > 20% are shown

### Surgeries performed

**Fig. 2:**
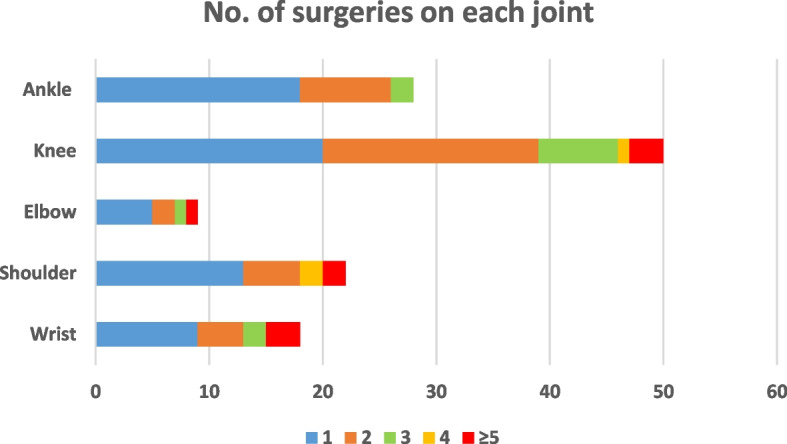
Number of surgeries performed on each joint for the 69 patients with EDS

Among the 69 patients, a total of 127 primary operations were carried out on one or more joints (Fig. 2). Mean age at primary surgery was 25.6 ± 11.1 years. Among the 69 patients, 50 (39.4%) primary surgeries were carried out on the knee, 28 (22.0%) on the ankle, 22 (17.3%) on the shoulder, 18 (14.2%) on the wrist and nine (7.1%) on the elbow. In addition to the 127 initial surgeries, 89 supplementary surgeries also took place: knee (*n* = 40), shoulder (*n* = 15), wrist (*n* = 14), ankle (*n* = 12) and elbow (*n* = 8). One-fifth of all operations (20.8%) were carried out by arthroscopy. At the time of primary surgery, the surgeon was alerted to the diagnosis or suspicion of EDS in 33.9% of cases.

### Shoulder surgery

Primary surgery was carried out on 22 shoulders, among which nine (40.9%) underwent more than one intervention (Table 2). Five patients underwent surgery on both shoulders. Mean age at primary shoulder surgery was 27.2 ± 13.8 years. There was a similar number of primary operations on the dominant and non-dominant sides (12 vs. 10, respectively). The main reason for carrying out surgery was anterior shoulder instability. Before surgery, 15 shoulders had been treated with physical therapy, five were treated with a compression garment and 11 were supported with an orthosis. The diagnosis of EDS was known preoperatively in 59% of cases.

**Table 2:** Surgical procedures carried out on each joint as primary and secondary interventions (*n* = 69 patients)

Type of primary surgery (*n* = 127)	Type of secondary surgery (*n* = 89)
**Shoulder**	**Shoulder**
Coracoid bone block (11), Bankart – arthroscopic (4), arthroscopic bone block (2), posterior block (2), arthroplasty (2), capsuloplasty (1)	Iliac crest bone block (5), removal of screw (bone block fixation) (4), neurolysis (1), osteosynthesis (1), arthroplasty (1), Bankart – arthroscopic (1), surgical site infection (1), coracobrachialis tenodesis (1)
**Elbow**	**Elbow**
Osteosynthesis (5), ulnar nerve transposition (3), hygroma (1)	Ablation of material (4), osteosynthesis (2), transposition of nerve (1), tendon transfer for radial nerve palsy (1)
**Wrist**	**Wrist**
Arthroscopy (TFCC/SL) (5), wrist arthrodesis (4), osteosynthesis (4), removal of cyst (4), capsuloplasty (1)	Ablation of material (5), osteosynthesis (3), SL repair (3), arthroscopy (TFCC/SL/cyst) (2), removal of cyst (1)
**Knee**	**Knee**
Reduction or transposition of the TTA (14), arthroscopy for meniscus procedure (12), stabilisation of the patella (other procedures) (4), ACL ligamentoplasty (± meniscus procedure) (4), realignment osteotomy (3), reinsertion of the patellar tendon (2), arthroscopy for osteochondral lesions (2), osteosynthesis (1), proximal tibio-fibular arthrodesis (1)	Ablation of material (14), repeat stabilisation of the patella (9), arthroscopy for meniscal procedure (5), ACL ligamentoplasty (3), osteosynthesis (2), arthroscopy for osteochondral lesions (2), LCL reinsertion (1), lateral tenodesis (Lemaire) (1), realignment osteotomy (1), infection (1), other (1)
**Ankle**	**Ankle**
Ligamentoplasty (14), osteosynthesis (6), reconstruction of the fibular retinacula (3), freeing of the tarsal canal (2), arthroscopy for OLAD (1), club foot (1), reinsertion of the Achilles tendon (1)	Further ligamentoplasty (6), ablation of material (2), epiphysiodesis (1), Achilles tendon debridement (open procedure) (2), arthroscopic debridement (1)

*TFCC* triangular fibrocartilage complex, *SL* scapholunate ligament, *TTA* tibial tuberosity advancement, *ACL* anterior cruciate ligament, *LCL* lateral collateral ligament, *OLAD* osteochondral lesions of the talus (astragalus) dome

Perioperatively, the shoulders were less painful when general anaesthesia (GA) was combined with local or regional anaesthesia (LRA) (mean VAS: 3.9 vs. 4.9, respectively). However, LRA was ineffective in 36.4% of cases. Primary surgery was carried out arthroscopically in six cases. Postoperatively and over time, 72.7% of the shoulders dislocated, independently of the dominant or non-dominant side. However, the dominant shoulders became painful more frequently than the non-dominant shoulders (91.7% vs. 70%, respectively). Seven patients needed to wear a splint or compression garment all the time and this always concerned surgery on the dominant side. One case of capsulitis and two cases of complex regional pain syndrome (CRPS) were reported. The types of surgical procedure carried out on the shoulder as primary or secondary intervention are summarised in Table 2.

After a longer follow-up period, the dominant shoulders had worse Quick-DASH scores than the non-dominant shoulders, and these scores were worse with the number of additional surgeries. A similar trend was observed for the SSV and VAS pain. Overall, 41.7% of patients were satisfied or very satisfied with the surgery performed on their dominant side and 70% on their non-dominant side (Table 3).

**Table 3:** Functional scores after shoulder surgery

Functional score	Dominant shoulder (*n* = 12)	Non-dominant shoulder (*n* = 10)
Quick-DASH score (mean)	58.14 ± 19.25	48.86 ± 30.70
Quick-DASH after 1 surgery	46.59 ± 13.69 (*n* = *6*)	40.58 ± 29.88 (*n* = *3*)
Quick-DASH after > 1 surgery	69.70 ± 17.53 (*n* = *6*)	68.18 ± 27.65 (*n* = *3*)
SSV (mean)	49.2 ± 18.3	57.9 ± 30.2
SSV after 1 surgery	56.7 ± 16.3	65.8 ± 18.5
SSV after > 1 surgery	41.7 ± 18.3	41.3 ± 50.0
VAS pain (mean)	4.8 ± 2.4	3.6 ± 3.1
Satisfaction		
Satisfied/very satisfied	5 (41.7)	7 (70.0)
Somewhat satisfied/dissatisfied	7 (58.3)	3 (30.0)

Values shown are mean ± standard deviation or n (%)

*SSV* Subjective shoulder Value

### Elbow surgery

A total of nine elbows underwent surgery, among which four were operated on more than once (44.4%) (Table 3). A single patient underwent surgery on both elbows, due to ulnar nerve instability. Mean age at the time of primary surgery was 24.2 ± 14.2 years. The non-dominant side was operated on more frequently than the dominant side. The main reason for primary surgery was elbow fractures. Before surgery, two elbows received functional treatment with orthoses and one underwent physical therapy. The diagnosis of EDS was known preoperatively in only 22% of cases.

Perioperatively, six elbows were operated on under GA only and three with LRA only. LRA was badly tolerated in two-thirds of cases, but the patients did not report having received supplementary GA. No arthroscopic procedure was performed.

Postoperatively, the dominant elbows had poorer Quick-DASH scores than the non-dominant elbows. This improved with the number of surgeries performed (Table 4) and the elbow became a less painful joint (VAS: 0.8 ± 0.8). All patients were satisfied or very satisfied with the procedures carried out on the dominant side and 83.3% on the non-dominant elbow. After surgery, only one patient needed to wear a brace on their dominant operated side. Postoperatively and over time, one elbow became unstable, two were stiff and two remained painful. The surgical procedures carried out as primary surgery and secondary interventions are summarised in Table 2.

**Table 4:** Functional scores for the dominant and non-dominant elbows after surgery

Functional score	Dominant elbow (*n* = 3)	Non-dominant elbow (*n* = 6)
Quick-DASH (mean)	42.42 ± 33.12	36.74 ± 21.53
Quick-DASH after 1 surgery	56.82 *(n* = *1)*	38.07 ± 18.68 *(n* = *4)*
Quick-DASK after > 1 surgery	35.23 ± 43.39 *(n* = *2)*	34.09 ± 35.36 *(n* = *2)*
VAS (mean)	1.0 ± 1.0	0.7 ± 0.8
Satisfaction		
Satisfied/very satisfied	3 (100)	5 (83.3)
Somewhat satisfied/dissatisfied	0 (0)	1 (16.7)

Values shown are mean ± standard deviation or n (%)

### Wrist surgery

Eighteen wrists were operated on, among which nine underwent surgery more than once (50%) (Table 2). Four patients underwent surgery on both wrists. Mean age at primary surgery was 27.9 ± 11.6 years. The most common reason for wrist surgery was scapholunate instability. Before surgery, five wrists had undergone physical therapy, two were treated with compression garments and eight were supported with orthoses. The diagnosis of EDS was known preoperatively in only 39% of cases.

Perioperatively, the wrists were less painful when GA was combined with LRA (VAS: 2.0 ± 1.4 vs. 2.4 ± 2.2 for GA alone, respectively). Nine wrists were operated on under LRA only and the pain in these cases was more important than in the other groups (VAS: 3.3 ± 2.8). LRA was ineffective in 27.8% of cases, irrespective of whether it was combined with GA or not.

Postoperatively and over time, the non-dominant wrists had better Quick-DASH scores than the dominant wrists, except in cases when several operations had been performed (Table 5). As with the shoulder, the dominant wrist became more painful than the non-dominant wrist (60% vs. 37.5% of cases, respectively). Two patients complained of instability of the wrist and one of stiffness. Six patients continued to wear an orthosis, particularly on the dominant side. Finally, 70% of patients were satisfied or very satisfied after surgery on their dominant side and 87.5% on their non-dominant side. The procedures carried out on the wrists are summarised in Table 2. Arthroscopy was performed in five cases for their first surgery and in two cases for additional procedures.

**Table 5:** Functional scores for the dominant and non-dominant wrists after surgery

Functional score	Dominant wrist (*n* = 10)	Non-dominant wrist (*n* = 8)
Quick-DASH (mean)	39.55 ± 23.58	34.66 ± 19.62
Quick-DASH after 1 surgery	48.86 ± 17.71 (*n* = *6*)	28.03 ± 11.66 (*n* = *3*)
Quick-DASK after > 1 surgery	25.57 ± 26.66 (*n* = *4*)	38.64 ± 23.51 (*n* = *5*)
VAS (mean)	3.0 ± 3.1	2.0 ± 2.1
Satisfaction		
Satisfied/very satisfied	7 (70)	7 (87.5)
Somewhat satisfied/dissatisfied	3 (30)	1 (12.5)

Values shown are mean ± standard deviation or n (%)

### Knee surgery

Fifty knees underwent surgery, among which 30 were operated on more than once (60%) (Table 2). Twelve patients underwent surgery on both knees. Mean age at the time of primary surgery was 23.9 ± 9.5 years. The main reason for knee surgery was patellar instability (50%). Preoperatively, 34 knees underwent physical therapy, five were treated with a compression garment and 27 were supported with an orthosis. The diagnosis of EDS was known preoperatively in only 22% of cases.

Primary surgery was carried out by arthroscopy in 36% of cases. The majority of patients (*n* = 44; 88%) underwent surgery under GA only. In six cases, LRA or spinal anaesthesia were performed. In two cases, the anaesthetist had to repeat spinal anaesthesia. The combined use of GA and spinal anaesthesia helped to reduce pain (VAS: 3.7 ± 3.1 vs. 4.3 ± 3.0 for spinal anaesthesia only). Despite surgery, 58% of patients continued to need a mobility aid and among these 11 patients walked with one or two crutches. Mean Lysholm-Tegner score was 63.7 ± 19.7. This was better for patients who underwent surgery once than for patients who underwent several operations (72.4 ± 22.6 vs. 57.8 ± 15.3, respectively). In 62% of cases, lameness persisted. Pain was constant after surgery in 16% of cases (VAS: 3.7 ± 3.1). In total, 70% of patients were satisfied or very satisfied with their surgery. Postoperatively and over time, 40% of patients reported instability of the knee. The operations were complicated by deep vein thrombosis (*n* = 1), sepsis (*n* = 1), CRPS (*n* = 2) and dysesthesia around the scar (*n* = 3). The surgical procedures carried out as primary and secondary surgery are shown in Table 2.

### Ankle surgery

Surgery was carried out on 28 ankles, which 10 were operated on more than once (35.7%) (Table 2). Seven patients underwent surgery on both ankles. Mean age at primary surgery was 26.5 ± 10.4 years. The main reason for surgery was ankle instability (50%) followed by ankle fractures. Arthroscopy was rarely used. Before surgery, 17 ankles were treated by physical therapy, four used a compression garment and 15 were supported with an orthosis. The diagnosis of EDS was known preoperatively in only 39% of cases.

The majority of patients underwent surgery under GA (*n* = 18; 64.3%). In terms of pain, there was a greater benefit from GA only compared to GA + LRA or spinal anaesthesia (mean VAS: 2.4 ± 2.2 vs. 5.5 ± 3.5 and 4.4 ± 3.5, respectively). In one case, the anaesthetist had to repeat the spinal anaesthesia.

Despite surgery, 64.3% of patients continued to need a mobility aid and among these, six patients walked with one or two crutches and eight always wore an orthosis. In terms of EFAS score, most patients had difficulty walking on uneven ground, could not run and had to modify their sporting practices. Overall, 75% of patients were satisfied or very satisfied with their surgery. Over time, 14% of patients reported persistent ankle instability. The operations were complicated by CRPS (*n* = 1) and dysesthesia around the scar (*n* = 2). The surgical procedures performed as primary and secondary surgery of the ankle are shown in Table 2.

## Discussion

This is the first descriptive study of the results of surgery in patients with non-vascular EDS. It is also the largest series of patients with a confirmed diagnosis of EDS and known functional scores undergoing orthopaedic surgery. Our results show that surgery for joint instability has a greater chance of success when it is carried out in patients where the diagnosis of EDS is known preoperatively. Postoperatively, there was a high rate of dislocations in these patients (72.7%), but a low rate of reoperations, with the majority of patients (> 70%) being satisfied with their surgery. In addition, our study also demonstrates that arthroscopic procedures have an important role to play in these patients, particularly when surgery is performed on the knee.

To date, a major part of the literature regarding joint instability in non-vascular EDS consists of case reports or technical notes, mainly concerning the shoulder or knee. Stanitski et al. described the orthopaedic manifestations of EDS and their functional impact without analysing the specific effects of surgery in patients with EDS [14]. In a recent study, shoulder arthroplasty (SA) in patients with EDS was a viable option and gave similar results to SA in patients with osteoarthritis or cuff tear arthropathy in terms of postoperative pain, range of motion, complications and reoperations [15]. For Tibbo et al. the improvement in function of patients with EDS after primary total knee arthroplasty (TKA) was similar to that of patients undergoing TKA for osteoarthritis, with no significant difference in reoperation or revision rates between the two groups (*p* < 0.05) [16]. Regarding total hip arthroplasty (THA), Guier et al. showed a significant improvement in postoperative Harris Hip Scores after THA in patients with EDS, but these patients had a high rate of dislocation after surgery [17]. A high rate of dislocation after THA and worse implant survival at 5-years was also reported in patients with EDS by Moore et al. [18].

In our survey, surgical intervention was uncommon in patients with EDS (only 236 patients underwent surgery out of 1368 (17.3%) followed in the reference centre). Furthermore, only 75 (31.8%) of the 236 patients undergoing surgery had a diagnosis of EDS according to the new 2017 EDS criteria (Fig. 1). A preoperative diagnosis of EDS was known for only 22% of patients undergoing surgery on the knee or elbow whereas this figure was 59% for patients undergoing shoulder surgery. The benefit of surgery appears to be maximal in patients where instability is linked to a preoperative diagnosis of EDS and the failure of joint surgery in a young person with EDS should suggest a diagnosis of EDS.

In the general population, instability of the shoulder is classically treated surgically by coracoid bone block or a Bankart repair depending on the background of the patient (age, sporting activity) and assessment of the lesions by imaging. In patients with non-vascular EDS, the shoulder should be treated initially with specific physical therapy exercises, focussing on dynamic kinetics, resting rotator cuff tone and scapulothoracic mechanics, before resorting to surgery [19]. In our series, nine shoulders required two or more reinterventions (40.9%). Out of the four initial Bankart repairs, one shoulder was reoperated on multiple times for a bone block procedure, which ultimately failed. No article in the literature has addressed the results of a Bankart repair in patients with EDS and most articles describe case reports or surgical techniques only [19]. In a series of 15 patients (20 shoulders), including five with EDS and 10 with hyperlaxity syndromes without a genetic diagnosis, Dewing et al. demonstrated the interest of anterior capsule-labral reconstruction for recurrent shoulder instability with an improvement in functional scores and the absence of repeat dislocation in 45% of cases at 3.8 years [20]. In another study of five shoulders (four patients) with EDS, open capsular shift combined with Achilles allograft augmentation of the anterior capsule restored shoulder stability in 4/5 (80%) shoulders and decreased pain (VAS: 7 preoperative vs. 2 at last follow-up), with only one case of revision surgery for recurrent posterior shoulder instability after an injury 1.6 years after the initial surgery [21]. Despite the high risk of complications, shoulder surgery can be proposed in patients with non-vascular EDS, including a coracoid bone block procedure, (known as Latarjet) after failure of medical therapy, in particular on the non-dominant shoulder where the best results are expected. Although we observed a higher risk of recurrence of shoulder dislocation postoperatively in patients with non-vascular EDS than in the general population (72.7% in this series vs. < 5% in the general population), the rate of reinterventions for dislocation after a primary Latarjet procedure was 2.7% (*n* = 3/11) in this study vs. 1.6% in the literature [22].

The knee was the joint that most frequently required more than one intervention (30/50; 60% of knees) (Table 2). Patellar instability was the most frequent cause of primary knee surgery (62%). However, it was also the primary cause of reinterventions (60% of cases; *n* = 30/50) for failure of stabilisation or ablation of material. Reconstruction of the medial patellofemoral ligament has mainly been studied in patients with JHS. These patients are improved by this type of patellar stabilisation, but the functional result is less satisfactory than in the general population [23]. In this series of 25 patients with hypermobility defined according to Beighton criteria, patellar stabilisation by reconstruction of the medial patellofemoral ligament resulted in a significant improvement of function, but this was significantly less than in control patients (*p* < 0.01). The authors concluded that joint hypermobility is not a contraindication to reconstruction of the medial patellofemoral ligament, but the expectations of patients with hypermobility should be managed carefully before considering surgery [23]. In our opinion, this surgical solution does not appear to be an option for the treatment of patellar instability in patients with non-vascular EDS. No other study has reported the results of anterior tibial tuberosity transfer in this population. Questions on the nature of the graft to be used for anterior cruciate ligament (ACL) repair have been raised in patients with tissue fragility. To our knowledge, there is no consensus on the choice of graft and the use of allografts has not been demonstrated specifically. Good results have been reported with ACL reconstruction for patellar instability in patients with hypermobility and EDS [24–27]. In our series, reconstruction was most often performed using modifications of the technique originally described by Dr Kenneth Jones [28] and no repeat ligamentoplasties were necessary. The ligamentoplasties performed in patients undergoing two or more operations were carried out after an initial meniscal procedure for patellar stabilisation. In patients with JHS, reconstruction of the ACL using a bone-tendon-bone graft gave the best results in terms of residual laxity and functional score compared to ligamentoplasty using the hamstrings [27]. By extrapolation, and in light of our results, reconstruction of the ACL using the Kenneth Jones technique appears to be good option in patients with hEDS.

In the current series, surgery to the elbow was usually secondary to fractures or due to ulnar nerve instability. Overall, joint instability was not a cause for surgery. Granata et al. demonstrated a high prevalence of ulnar nerve instability of the elbow in patients with hSED [29]. There are two hypotheses for this: (i) an increased frequency of peripheral neuropathies [30]; and (ii) anatomic variation of the Osborne ligament, which is looser in patients with hEDS [31]. In terms of residual pain, DASH score and patient satisfaction, endoscopic decompression has been shown to give similar results to decompression with anterior transposition in idiopathic cubital tunnel syndrome [32]. In our series, all patients underwent nerve transposition by open surgery although endoscopic-assisted subfascial anterior transposition of the ulnar nerve has been reported to be a more refined technique with good functional outcomes [33]. Patients with EDS often present with coagulation problems and low body weight due to gastrointestinal and eating problems [34, 35]. In a comparative study of open versus endoscopic cubital tunnel release, Buchanan et al. reported equivalent overall clinical improvement after the two types of procedure, but patients in the endoscopic group had a significantly higher incidence of postoperative haematoma (OR = 5.70 [95%CI: 1.2–27.03; *p* = 0.03) [36]. Therefore, extra care should be taken in patients with hEDS due to the increased risk of haematoma in this group and the theoretical contraindications to cubital tunnel release in underweight patients [36]. Finally, there was a low reoperation rate on the elbow of 44.4% (4/9 elbows).

In the current series, wrist surgery was usually carried out for scapholunate instability or pathologies associated with the distal radioulnar joint. The high number of palliative surgeries (represented by wrist arthrodesis) suggests that surgical management of these instabilities is difficult. No parallel study is available in the literature and only case reports have been published. The use of allografts for ligamentoplasty has been reported for instability of the extensor carpi ulnaris with good results [37]. The rate of reinterventions on the wrist in the present study was 50%.

Surgery was performed on the ankle in 28 cases. Ligamentoplasty was the most frequent procedure performed. This was also the leading cause of reoperations, which occurred in 35.7% of cases (10/28). One of the reasons for this is probably graft failure due to the poor quality of the tissues. In these cases, it would probably be more beneficial to use an allograft. No recommendations are available regarding the surgical management of chronic ankle instability in patients with non-vascular EDS.

The management of anaesthesia may be difficult in these patients. In our survey, 42% experienced problems with GA and 47% were insensitive to LRA. These results are consistent with data in the literature, which describe resistance to local anaesthetics, in particular lidocaine and bupivacaine [38].

To date, the current study is the largest survey specific to patients with hEDS and sheds light on the types of joint surgery performed in these patients. However, it has some limitations. It was retrospective in nature and the collection of data by video-consultation may have been responsible for inaccuracies in responses relating to surgical and anaesthesia histories. Furthermore, the absence of preoperative functional scores means that we could not evaluate any gains achieved postoperatively. Finally, a comparison with other patient populations without EDS would have helped to define the benefits or pitfalls of specific surgical procedures.

In conclusion, surgery for joint instability has the greatest chance of success when it is carried out in patients with an established diagnosis of EDS. It should be proposed after medical therapies have failed, including orthoses, compression garments and physical therapy. Patient management should take place within a specialist multidisciplinary team. Compared to the high relapse rate of shoulder dislocations (72.7%), the reintervention rates were low (35.7–60%) and the majority of patients (> 70%) were satisfied or very satisfied with their surgery. Although surgery has historically been avoided in patients with EDS and the rates of surgery remain low (17.3% in our cohort), surgical procedures may be beneficial in patients with non-vascular EDS and should be proposed with the aim of reducing pain and improving quality of life. Arthroscopy has an important role to play, particularly in knee surgery, and future case–control studies will be useful to confirm the benefit of arthroscopic procedures in non-vascular EDS patients.

## Data Availability

The data that support the findings of this study are not openly available due to reasons of sensitivity but are available from the corresponding author upon reasonable request. Data are located in controlled access data storage of “C2R Épidemiologie”, approved by the French Ministry.

## Abbreviations

ACL: Anterior cruciate ligament
BMI: Body mass index
CRPS: Complex regional pain syndrome
EDS: Ehlers-Danlos syndrome
EFAS: European Foot and Ankle Society
ENT: Ear, nose and throat
GA: General anaesthesia
hEDS: Hypermobile Ehlers-Danlos syndrome
LCL: Lateral collateral ligament
LRA: Local or regional anaesthesia
JHS: Joint hypermobility syndrome
NV-EDS: Non-vascular Ehlers-Danlos syndrome
OLAD: Osteochondral lesions of the talus (astragalus) dome
SA: Shoulder arthroplasty
SD: Standard deviation
SL: Scapholunate ligament
SSV: Subjective shoulder value
TFCC: Triangular fibrocartilage complex
THA: Total hip arthroplasty
TKA: Total knee arthroplasty
TTA: Tibial tuberosity advancement
VAS: Visual analogue scale
